# Analysis of convective and diffusive transport in the brain interstitium

**DOI:** 10.1186/s12987-019-0126-9

**Published:** 2019-03-06

**Authors:** Lori Ray, Jeffrey J. Iliff, Jeffrey J. Heys

**Affiliations:** 1 Chemical and Biological Engineering, Montana State University, Bozeman, MT USA; 2 Department of Anesthesiology and Perioperative Medicine, Oregon Health & Sciences University, Portland, OR USA; 3 Knight Cardiovascular Institute, Oregon Health & Sciences University, Portland, OR USA

**Keywords:** Biotransport, Parenchyma, Bulk flow, Finite element model, Real time iontophoresis

## Abstract

**Background:**

Despite advances in in vivo imaging and experimental techniques, the nature of transport mechanisms in the brain remain elusive. Mathematical modelling verified using available experimental data offers a powerful tool for investigating hypotheses regarding extracellular transport of molecules in brain tissue. Here we describe a tool developed to aid in investigation of interstitial transport mechanisms, especially the potential for convection (or bulk flow) and its relevance to interstitial solute transport, for which there is conflicting evidence.

**Methods:**

In this work, we compare a large body of published experimental data for transport in the brain to simulations of purely diffusive transport and simulations of combined convective and diffusive transport in the brain interstitium, incorporating current theories of perivascular influx and efflux.

**Results:**

The simulations show (1) convective flow in the interstitium potentially of a similar magnitude to diffusive transport for molecules of interest and (2) exchange between the interstitium and perivascular space, whereby fluid and solutes may enter or exit the interstitium, are consistent with the experimental data. Simulations provide an upper limit for superficial convective velocity magnitude (approximately $v$ = 50 μm min^−1^), a useful finding for researchers developing techniques to measure interstitial bulk flow.

**Conclusions:**

For the large molecules of interest in neuropathology, bulk flow may be an important mechanism of interstitial transport. Further work is warranted to investigate the potential for bulk flow.

**Electronic supplementary material:**

The online version of this article (10.1186/s12987-019-0126-9) contains supplementary material, which is available to authorized users.

## Background

Transport of interstitial molecules is an essential link in many physiological processes of the brain. For example, transport governs the dynamics of physiologically-active molecules, including extra-synaptic signaling of neuromodulators, and the dynamics of pathological molecules that transit the extracellular space (ECS) [1]. The mis-aggregation of intracellular and extracellular proteins is a common feature of neurodegenerative diseases, including the formation of extracellular plaques comprised of amyloid β (Aβ) in Alzheimer’s disease. The clearance of Aβ, a soluble, interstitial peptide that is released in response to synaptic activity, is impaired in the aging and the Alzheimer’s brain, and the impairment in the clearance of mis-aggregating proteins is believed to underlie the vulnerability of the aging and injured brain to the development of neurodegeneration [2, 3]. Understanding mechanisms of solute transport in the brain has fundamental and wide-ranging applications.

Controversies exist regarding the relative importance of diffusive versus convective solute transport in the brain interstitium [4–7]. In this work, we describe a tool developed for investigating interstitial transport mechanisms, where the contributions of diffusive and convective transport can be quantified and explored for molecules of interest. In addition, the tool is used to investigate the nature of transport between perivascular and interstitial space.

### Physiology of the brain interstitium

Despite the incredible complexity of the brain, transport of molecules within brain tissue has been successfully described using relatively simple models. Brain tissue is comprised of cells (including cell bodies and processes, neurons and glia) along with the extracellular space (ECS) between cells. The ECS is a continuously-connected network filled with interstitial fluid (ISF), where interstitial transport occurs. In addition to being fluid-filled, an important constituent of the ECS is the extracellular matrix consisting of proteins [8].

Brain tissue is penetrated by vasculature, supplying nutrients to the cells; however, within the brain this exchange is strictly controlled and limited by the blood–brain-barrier (BBB). Researchers have established the presence of an annular space surrounding the penetrating vasculature, the perivascular space (PVS), that is connected to subarachnoid cerebrospinal fluid (CSF), providing a potential source of interstitial fluid and efflux route for interstitial solutes and fluid [9]. The exact make-up of the PVS is under investigation with two main theories: (1) a fluid-filled space between the vessel walls and endfeet (possibly containing connective tissue) and (2) perivascular pathways via basement membranes [7].

**Fig. 1:**
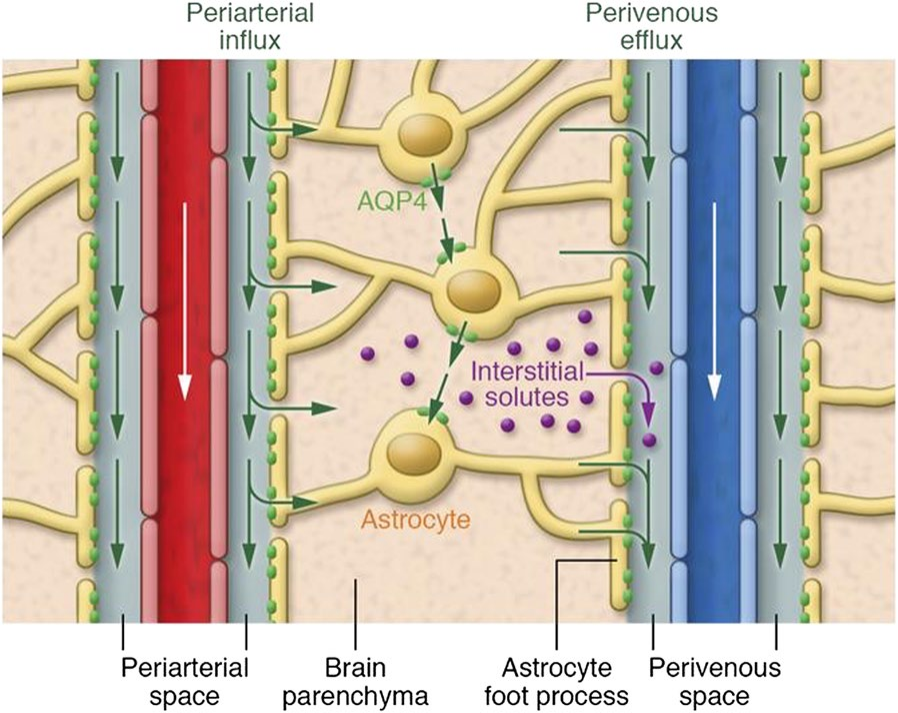
Illustration of movement of fluid and solutes in brain tissue between interstitial tissue (parenchyma) and perivascular space surrounding penetrating vasculature. Green arrows indicate fluid transport, whether by diffusion, dispersion, or convection and diffusion has not been established. The figure shows movement of fluid along the periarterial space into the interstitium and out along the perivenous space. This is one proposed theory, and other evidence suggests periarterial and perivenous transport in the opposite direction of blood flow. Purple indicates interstitial solutes; solutes exit the interstitial space through gaps in the astrocytic endfeet to either to the perivenous or periarterial space, where they are cleared to primary para-venous drainage pathways or the CSF. Although the interstitial space looks essentially open in this illustration, it is crowded with cells and extracellular matrix where both fluid and solutes move along a tortuous path in a restricted extracellular liquid volume that comprises approximately 20% of the total volume

The PVS is surrounded by a sheath of astrocytic endfoot processes (astrocytes are glial cells with several long cellular processes terminating in endfeet, see Fig. 1). To enter or exit the ECS via the PVS, molecules must pass through the gaps between the endfeet (Fig. 1). We will term this sheath of overlapping processes the ‘perivascular wall’ (PVW). There is conflicting evidence for both the coverage of the vessel by these endfeet and the size of the gaps. Mathiisen et al. analyzed rat electron microscopy (EM) images of the perivascular astroglial-sheath prepared by chemical fixation, measuring the gaps at 24 nm in a 1.5-μm thick (on average) wall and calculating 99.7% coverage of the PVW surface of capillaries [10]. In comparison, the ECS comprises 20% of brain tissue and typical channels are 40–60 nm in width [11, 12]. Korogod et al. found the coverage to be 94.4% using chemical fixation and 62.9% using cryo fixation [13]. The cryo fixation result of 37% extracellular space is even grater than the ECS void volume, suggesting that the PVW may present no barrier to transport of molecules. In addition, the endfeet contain protein channels that facilitate transport of specific molecules across the cell wall, such as the transport of water by aquaporin-4 (AQP4) channels.

Conflicting evidence has been presented regarding the presence of convection in the interstitium [4, 5, 11, 14], described further in “Experimental techniques for investigating brain transport”. Molecular exchange between perivascular spaces and the brain interstitium is clear from experimental observation [4, 5, 7]. Strong evidence exists for transport in the PVS that is more rapid than can be explained by diffusion, possibly transport by convective flow or dispersion [4, 5, 9, 11, 15, 16]. The direction of transport along perivascular spaces, with or against blood flow, is debated and both have been observed experimentally [4, 5, 7, 16–19]. Transport via perivascular routes is observed to be more rapid than transport through the interstitium [4, 5].

### Transport in biological tissues

Movement of molecules in the interstitial fluid occurs by two possible mechanisms: diffusion and convection. Diffusion occurs via the random motion of molecules; movement is from high to low concentration and depends upon the size of the molecule. Convection is the transport of a substance by bulk flow, where bulk flow is often the movement of fluid down a pressure gradient. In a free medium, convection is molecular-size independent; all solute molecules move in the direction and with the velocity of the bulk flow.

Applying the simplification of a stationary phase (the cells) and a mobile phase (the ISF), brain tissue is often characterized as a porous media, where void volume (α) and tortuosity (λ) describe the porous nature of the material [14]. Void volume is the fraction of the ECS volume to the total volume. Tortuosity represents the degree to which molecular transport is slowed by the porous medium; it is a property of both the medium and the molecule. Tortuosity incorporates: (1) the additional distance a molecule must travel to move around obstacles in the medium, including dead spaces (“dead-end” pores); and (2) how its progress is slowed by interaction with the walls and extracellular matrix, or exclusion from pathways due to molecular size. A void volume of about 20% and tortuosity of about 1.6 (for small molecules) are surprisingly consistent across brain regions and adult species (and likely reveal something about the most efficient ECS arrangement) [20].

Superficial velocity is used to characterize flow in porous media; it is a hypothetical flow velocity calculated as if the mobile (liquid) phase were the only phase present in a given cross-sectional area. Intrinsic velocity is the actual liquid velocity within the ECS at a specific location. Superficial velocity ($v$) is related to intrinsic velocity ($v_{i}$) through $v_{i} = v/\alpha$.

Using a porous media model requires an implicit assumption that the very heterogeneous properties of brain tissue average out over the scale of interest such that the medium behaves in a homogeneous manner. An exception to this assumption in the brain interstitium is the exchange between interstitial and perivascular space at discrete locations of the penetrating vasculature, where molecules may either enter or leave the interstitium. As penetrating vasculature is separated by approximately 175–280 μm [21, 22], a regular heterogeneity is introduced into tissue that can otherwise be treated as homogenous at the millimeter scale.

### Experimental techniques for investigating brain transport and their findings

Real-time iontophoresis (RTI) [23] is a quantitative experimental technique that is the gold-standard for investigating transport in brain tissue. A large body of data has been gathered from healthy adult brains in different regions and several species, both in vivo and in vitro, and these data form a critical reference set for all discussions of transport in the brain [14, 20]. In RTI, a small ionic molecule, commonly tetramethylammonium (TMA), is applied to brain tissue at a known rate using a 2–5 μm probe and its concentration measured over time at a point 100–200 μm away. RTI is limited to a few molecules, chosen for their lack of cellular interaction and ionic properties. The source is turned on for a time and then off, so both the rise and fall of concentration are measured and fitted to a model to obtain values for α and λ. Traditionally, a diffusion-only, homogenous porous media model is used, for which there is an analytical solution [23].

Although RTI (like many quantitative neuroscience experiments) is a difficult technique that requires extreme attention to detail and suffers from many sources of variability, surprisingly consistent and reliable data have been obtained. Sources of variability may include: tissue damage, inter-animal anatomic and physiologic variation, tissue heterogeneity, iontopheretic variations within living tissue, and experimental variations (such as differences in micropipette glass properties, weather, etc.). The distance between probes is measured (reported to the nearest micron) and accounted for in the data analysis. Table 1 provides a summary of RTI results from several sources, demonstrating both reproducibility across labs and around 1% standard deviation of the output parameters between experimental replicates.

**Table 1:** Summary of ECS structural parameters determined by TMA-RTI experiments on neocortex of healthy, anesthetized adult rat and mice (layer indicated in table)

Reference	Preparation	Void volume	Tortuosity
Mice			
Xie [24]	In vivo	0.227 ± 0.003	1.55 ± 0.05
Kress [25]	In vivo	0.224 ± 0.080	1.50 ± 0.11
Rat: cortex layers V and VI			
Lehmenkuhler [26]	In vivo	0.23 ± 0.005	1.59 ± 0.018
Vorisek [27]	In vivo	0.23 ± 0.02	1.55 ± 0.02
Mazel [28]	In vivo	0.22 ± 0.01	1.60 ± 0.02
Kume-Kick [29]	Brain slice	0.24	1.72
Rat: cortex layers II and III			
Lehmenkuhler [26]	In vivo	0.20 ± 0.003	1.57 ± 0.03
Cserr [30]	In vivo	0.18 ± 0.03	1.57 ± 0.05
Mazel [28]	In vivo	0.22 ± 0.01	1.61 ± 0.01
Perez-Pinzon [31]	Brain slice	0.18 ± 0.008	1.62 ± 0.039

Uptake constant omitted from table because it has not been observed to vary much over in vivo rat brain experiments, 0.003–0.006 s^−1^ [20]

**Fig. 2:**
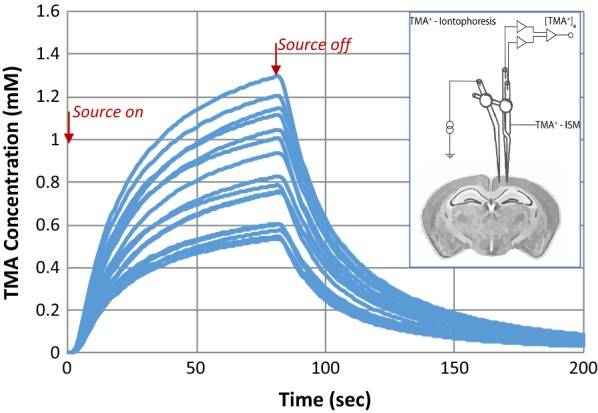
TMA concentration curves for each replicate of young adult mice from Kress [25], generated from data for void volume, tortuosity, and uptake using RTI equations from Nicholson [14]. The replicates demonstrate experimental variability, where range is 88% and the standard deviation in $C_{max}$ is 36%. The inset shows an RTI experimental set up, where source and detection probes are inserted into brain tissue. The source probe delivers molecules to the brain tissue; the detection probe measures the concentration of those molecules over time. Analysis of the resulting concentration curve provides an estimate of α and λ

Analysis of the data from RTI experiments to useful values describing the structure of the ECS has assumed diffusion-only transport and homogenous, isotropic tissue, including homogeneity with respect to cellular uptake, adsorption and physiological efflux (all contained in the “uptake” constant, k). Therefore, one might be tempted to take the success and reproducibility of these experiments as evidence that these assumptions are correct. However, upon reproducing experimental TMA concentration curves from data reported for each replicate (Fig. 2) one finds more variability inherent in the raw data. Significant spread or range is observed in the experimental curves where:

$$range = \left( {C_{max, high rep} - C_{max, low rep} } \right)/C_{max, mean}$$

where: *C*_*max*_ = the peak concentration in the TMA concentration curve, *C*_*max,high rep*_ = *C*_*max*_ for the highest experimental replicate, *C*_*max,low rep*_ = *C*_*max*_ for the lowest experimental replicate.

Replicates reported by Cserr et al. in rats, Xie et al. in mice and raw data obtained by the authors for individual replicates in mice presented in Kress et al., reveal consistent variability in reproduced TMA concentration curves—the range is 70–90% [24, 25, 30]. Although these three experiments represent a fraction of all RTI data, such a consistent experimental range leads one to question whether some physical phenomenon is being overlooked that may be revealed by analyzing the data using models different from diffusion-only in a homogeneous material.

Integrative Optical Imaging (IOI) was developed to study the brain-transport properties of large molecules [32]. In the IOI method, macromolecules carrying a fluorescent label are injected by a pressure pulse and their progress measured by fluorescence microscopy. Although conceptually simple, analysis of the measurements is complex as the CCD camera registers a two-dimensional image of a three-dimensional “cloud” of diffusing molecules. Thus reported intensities do not correspond to actual concentrations, but some form of projection that depends on the optical characteristics of the imaging system. Analysis of the data to determine tortuosity applies the same model of diffusion-only transport in a homogeneous material (void volume cannot be calculated by IOI, but is often assumed to be the same as for small molecules). Tortuosity generally increases with molecular size, however, molecular shape and flexibility also play a role. The majority of data is from brain slices. However, in vivo IOI became possible around 2006 and this body of data continues to grow. The success of the experimental techniques that rely upon a diffusion-only model (RTI and IOI) lends credence to the theory that bulk flow may not be important to molecular transport in the brain interstitium.

Microscopy is another tool used to study transport in the brain; it can be qualitative or semi-quantitative. In vivo injection of a tracer followed by ex vivo microscopic investigation of fixated tissue is a dependable, though coarse method. In a 1981 study, Cserr et al. injected radiolabeled tracers varying in size from 0.9 to 69 kDa into brain interstitium and measure their clearance rate over time. All molecules cleared at similar rates, supporting a convective-dominated model of transport [33]. Cserr noted the molecules followed “preferential routes”, possibly associated with vasculature. However, the experiments lacked the spatial resolution to resolve whether bulk flow was occurring throughout the brain interstitium or was restricted to the PVS.

More recently, Iliff et al. used in vivo two-photon laser scanning microscopy to follow clearance of different-sized tracers through the brain and reported indications of interstitial bulk flow [4]. Transport from the subarachnoid CSF down the periarterial space and into the brain interstitium was observed for three tracers of varying molecular size (3, 40, and 2000 kDa, the largest tracer did not enter the interstitium) moving at similar rates—Iliff interpreted the results as being caused by convective flow. Iliff et al. used ex vivo fixation to observe the tracers leaving the interstitium along large venous structures to primary para-venous drainage pathways. In studies that confirmed the findings from Cserr et al., Iliff and colleagues observed the clearance rate of interstitially delivered Dextran-10 (10 kDa) was identical to mannitol (380 Da) [4]. Smith et al. conducted experiments similar to those of Iliff et al., corroborating convective transport along perivascular pathways, but finding that transport in the ECS was consistent with pure diffusion [5]. However, Mestre et al. [6] demonstrated the choice of anesthesia and tracer injection by pressure pulse employed by Smith et al. may suppress CSF influx, resulting in hindered tracer transport in the ECS. Smith et al.’s photo-bleaching results supporting diffusion-only in the interstitium were not questioned.

Iliff et al. also observed a 70% reduction in mannitol clearance from *Aqp4* knockout (KO) mice compared to wild-type (WT) mice, hypothesizing that astroglial aquaporin-4 (AQP4) may support interstitial and facilitated solute transport. Smith repeated these experiments, but did not observe differences in clearance for *Aqp4* KO vs. WT mice. However, a recently published study concurred that CSF influx is higher in WT mice than in four different *Aqp4* KO lines; and demonstrated a significant decrease in tracer transport in KO mice and rats [6]. Further, the study established that anesthesia, age, and tracer delivery may explain the opposing results.

### Estimating interstitial bulk-flow

Diffusion is always occurring. Convection requires a driving force, such as a pressure gradient, to generate bulk flow. It is hypothesized that a small pressure difference exists between the periarterial and perivenular space [4, 34], providing a mechanism for bulk flow across the interstitium. Bulk flow velocity in porous media can be calculated using Darcy’s law $\left( {v = - k^{{\prime }} \left( {\nabla P} \right)} \right)$, where $k^{{\prime }}$ is hydraulic conductivity, $\nabla P$ is the pressure gradient and $v$ is the superficial velocity. Table 3 reports literature values for hydraulic conductivity in brain tissue, which range over two orders of magnitude. The pressure gradient is the difference in pressure between the periarterial and perivenular walls divided by the distance between them. This pressure gradient is unknown, but can be estimated. There are two schools of thought on the genesis of the pressure gradient: (1) hydrostatic pressure, originating from intracranial pressure of less than 10 mmHg peak-to-peak, and (2) hydrodynamic pressure, generated by arteriolar pulsation (65–100 mmHg maximum pressure) translating through the elastic vascular walls and bounded by the more rigid perivascular walls [34]. The hydrostatic pressure gradient in the brain is probably quite small, with an estimated upper limit of 1 mmHg mm^−1^ [35]. The hydrodynamic pressure gradient would be larger, but still much less than the arteriolar pressure. From the arteriolar pressure, hydrodynamic pressure would be reduced (1) through translation across the vascular wall and (2) by flow of ISF through possible restrictions in the periarteriolar wall (either aquaporin channels in the endfeet or gaps between endfeet). Therefore, at the periarteriolar wall just within the interstitium, the hydrodynamic pressure will be a small percentage of the arteriolar pressure and higher than the very low perivenular pressure.

### Published simulations

Published simulations of transport in the brain fall into three categories: (1) structural or geometric models [20], (2) compartment models [36], and (3) continuum transport models. Transport models are derived using conservation principles. Many transport models for biological tissues successfully use the porous media assumption [37]. Both Jin et al. [38] and Holter et al. [35] developed thorough transport models of interstitial flow through an extracellular matrix constructed based on the EM work of Kinney for the rat CA1 hippocampal neuropil [39]. Each adjusted the EM in different ways to increase the void volume of the ECS to match experimental values of around 20% (volume changes are known to occur during tissue preparation and embedding for EM). Jin calculated a hydraulic conductivity of 1.2 × 10^−6^ cm^2^ mmHg^−1^ s^−1^ and Holter a hydraulic conductivity of 2 × 10^−8^ cm^2^ mmHg^−1^ s^−1^. Holter, using a hydrostatic pressure assumption, predicted average intrinsic velocities of less than 1 μm min^−1^ (superficial velocities of less than 0.2 μm min^−1^). Jin’s model includes diffusion and convection of a solute, investigating pressure differences of 0–10 mmHg and concluding: (1) convection preferentially accelerates transport of large molecules, (2) pressure differences of > 1 mmHg are required for convection to augment transport, and (3) diffusion alone adequately accounts for experimental transport studies [38]. Jin et al. verified their model using visual comparisons to (1) Iliff’s two-photon microscopy data [4] and (2) Thorne’s IOI data [40] (both for 3-kD molecules). However, concentrations predicted from their 2D model are not a direct comparison to intensity measured in an IOI experiment where the 2D image is convoluted by the projection from the 3D “cloud” of molecules (see IOI above). Asgari et al. show diffusion-only solute transport in the interstitium is increased by periarteriolar dispersion over periarteriolar diffusion [15]; for an interstitial injection, dispersion results in a lower solute concentration at the PVW. Different injection scenarios are investigated and demonstrate agreement with previously opposing experimental observations, providing hypotheses for both influx and efflux along either the periarteriolar or perivenular route. Asgari et al. also compared solute transport for 20-nm and 14-nm astrocytic endfeet gaps, with the smaller gap leading to a significant reduction in transport and corresponding increase in interstitial solute concentration.

In summary, convective transport in the brain interstitium is under debate, with conflicting evidence in the literature. Experimental observations support the ability of molecules, below a certain size (2000 kDa), to move between perivascular spaces and the interstitium; we call this ‘perivascular exchange’. Strong evidence exists for transport along the perivascular space that is faster than diffusion, although observations conflict on the direction of movement in the PVS, with or against blood flow. However, transport of molecules between the interstitium and the perivascular space at penetrating vasculature is independent of the direction of PVS-fluid movement. In addition, there is a lack of relevant modeling of interstitial brain transport-mechanisms where quantitative published data exists with which to verify results and inherent assumptions.

The goal of this work is to present a model of transport in the brain interstitium that can be quantitatively compared with well-established experimental data, and can test current hypotheses of interest in brain transport. Although studies utilizing sophisticated microscopy or IOI may be more contemporary and offer details not elucidated by RTI (such as the movement of macromolecules), they do not provide sufficient (microscopy) or applicable (IOI) quantitative data with which to verify the model. This work focuses on RTI experiments, which provide a large body of reviewed and confirmed data, with significant and accessible quantitative substance. The model is used to investigate (1) the presence of bulk flow in the brain interstitium by applying diffusion-only and diffusion with convective bulk flow to transport-model simulations of RTI-TMA experiments, and (2) the effect of perivascular exchange on the same.

### RTI experiments in the context of interstitial bulk flow

Although RTI experiments originally relied upon a diffusion-only model, recent research findings encourage investigating the potential for bulk flow in the interstitium between periarterial and perivenous spaces. Therefore, let us perform a thought experiment with these in mind. In an RTI experiment, two probes are inserted into the brain approximately 150 μm apart (Fig. 2 insert). The first (source) probe delivers molecules to the brain tissue; the second (detection) probe measures the concentration of those molecules over time. In an isotropic, diffusion-only model, the concentration is symmetric in space—it is the same in any direction at a given distance from the source. In a convective flow field however, the concentration would vary depending on the orientation of the path from source to detection point relative to the flow field. If the solute is diffusing in the same direction as the convective flow, a molecule moving away from the detection probe would be carried away more rapidly by the bulk flow, resulting in less accumulation and a lower maximum concentration. If the solute is diffusing against the convective flow, any solute randomly diffusing away from the detection probe would be carried back by bulk flow, resulting in greater accumulation and an overall increase in concentration. Since it is unlikely experimentally to align the probes with any potential flow field, there would most likely be a random sampling of orientations relative to the postulated flow field as each RTI test is performed, resulting in spread or range in the experimental data if bulk flow was present. As we will show using the model, larger bulk flows result in higher range and lower bulk flows or the absence of bulk flow results in lower range. Reciprocally, larger experimental range opens the potential for higher bulk flows being theoretically possible, and lower experimental range would imply a limit on the magnitude of any possible bulk flow.

## Methods

**Fig. 3:**
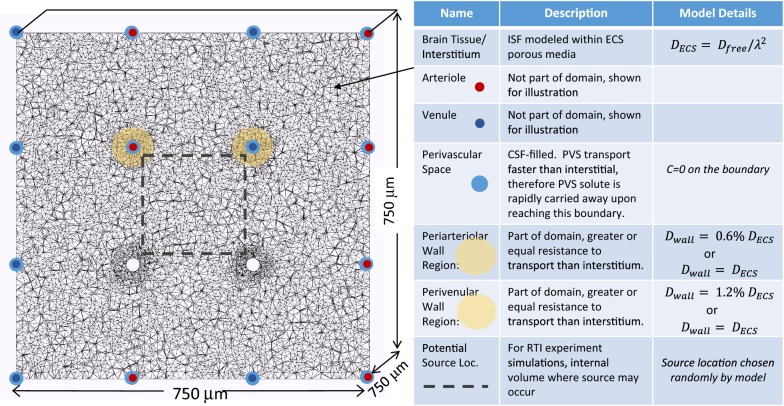
Finite-element domain illustrating physiology incorporated into model (2-dimensional slice of 3-dimensional domain). Cubic domain measures 750 μm on a side (0.4 mm^3^) with 8 penetrating arterioles and 8 penetrating venules. Red dots mark arterioles. Dark blue dots mark venules. Light blue annulus shows perivascular space that is connected to subarachnoid CSF. Yellow annulus marks the perivascular wall region, which may have a very low void volume resulting in slower mass transport than the bulk of the brain ECS. An arteriole and a venule are shown without shading to reveal the refinement of the mesh at these internal boundaries. The table contains additional information relating brain physiology to model parameters and boundary conditions. The 3-dimensional model uses a tetrahedral mesh of approximately 880,000 elements

A finite-element model of transport in the brain interstitium was developed based on porous-media flow and mass-transport equations. The model domain is a three-dimensional section of the interstitium with penetrating vasculature (eight arterioles and eight venules, typically). Figure 3 shows a two-dimensional slice of the domain where shading illustrates the PVS and PVW and the table relates the physiology to aspects of the model. Several model domains were tested to determine the size and shape that minimized the effect of the exterior boundaries on the simulation results. The potentially slower mass transfer through the perivascular wall is modelled as a narrow region surrounding each vessel where the diffusivity is a percentage of interstitial diffusivity. The PVS becomes a boundary of the model domain, where exchange between the PVS and the interstitium is modeled through the application of boundary conditions to the vessel walls.

The ISF is assumed to be an incompressible Newtonian fluid, and the brain tissue is assumed to exhibit porous media flow behavior. The flow velocity is modeled using Darcy’s law:

1 $$v = - k^{{\prime }} \left( {\nabla P} \right)$$

combined with steady-state mass conservation:

2 $$\nabla \cdot v = 0$$

where $v$ is the superficial velocity, $k^{{\prime }}$ is the hydraulic conductivity, and $P$ is the pressure. An oscillatory pressure is applied at the periarteriolar walls (different pressure magnitudes are explored and specified for each result), simulating physiological arteriolar pulsations. A pressure of zero is assumed at the perivenular walls. On the remaining exterior boundary, a symmetry assumption is used. Hydraulic conductivity is assumed to be homogeneous and isotropic. The distance between penetrating vessels varies by vessel size and location within the brain, and also by species. Here we are interested in the average distance between a distal penetrating arteriole and the nearest post-capillary venule in the rat neocortex. A value of 250 μm (center-to-center) is used based on limited anatomical data and values employed in similar models (see Table 2). To summarize results, the simulated superficial-velocity is averaged both in space and time; the spatial average is a volume-weighted average over the entire domain.

**Table 2:** Model parameters and variables

Parameter	Value	References
All simulations		
Arteriole diameter	30 μm	Iliff [34], Xiong [44], Mestre [16] (40 μm); other modeling studies: Jin [38], Holter [35]
Venule diameter	30 μm	Iliff [34], Xiong [44]; other modeling studies: Jin [38] (40 μm), Holter [35]
Flow simulations		
Vessel separation (distance between arterioles and venules)	250 μm	Nishimura [22] (175 μm rats), Adams [21] (280 μm primates), other modeling studies: Jin [38] (250 μm), Holter [35] (280 μm)
RTI and clearance simulations		
RTI current	From appropriate experiment	Cserr [30], Kress [25], Xie [24]
Free diffusivity	As reported for each molecule in “Methods” or “Results”	Nicholson [14]
Void volume	Mean value from appropriate experimental data	Cserr [30], Kress [25], Xie [24]
Tortuosity	Fitted. Increased from experimental mean value to fit RTI data. See “Discussion” of adjusted tortuosity	Cserr [30], Kress [25], Xie [24]

Variables	Value range	References
Flow simulations		
Hydraulic conductivity	2 × 10^−6^ to 2 × 10^−8^ cm^2^ mmHg^−1^ s^−1^	See Table 3
Pressure at arteriolar PVW	0.2 to 8 mmHg	See Table 3
RTI and clearance simulations		
Bulk flow velocity	0 to 250 μm min^−1^	By adjusting hydraulic conductivity and pressure within above ranges
PVW diffusivity	0.6 to 100% of D_ECS_	Mathiisen [10], Korogod [13], (see “Methods”)
Vessel separation (distance between arterioles and venules)	225–275 μm	Nishimura [22] (175 μm rats), Adams [21] (280 μm primates), other modeling studies: Jin [38] (250 μm), Holter [35] (280 μm)

Parameters are held constant; they are either taken from literature or fitted from experimental data. Variables are varied to test different transport hypotheses (see Table 4)

The mass transport equations modified for porous brain tissue are based on Nicholson and Phillips [14, 23]:

3 $$\frac{\partial c}{\partial t} = D^{*} \nabla^{2} c + \frac{s}{\alpha } - f\left( c \right) - v \cdot \nabla c$$

where: $c$ = concentration in the ISF, $D^{*}$ = apparent diffusivity = D/λ^2^, $s$ = source term, $\alpha$ = void volume = V_ECS_/V_total_, $f\left( c \right)$ = uptake term, assumed to be zero for simulations performed here (TMA was chosen as a probe because it exhibits no cellular uptake).

A solute may exit through either the periarteriolar or perivenular walls. As transport in the PVS is known to be much faster than in the interstitium [4, 5], it is assumed that upon reaching the PVS a solute is rapidly transported away. Note that no assumption about the direction of perivascular transport is required, only that it is rapid relative to interstitial transport. Therefore, a boundary condition of $c = 0$ is used on the vessel walls (see Fig. 3). For the perivascular walls, both tight, as observed by Mathiisen [10], and loose, as observed by Korogod [13], arrangements were considered. For the tight PVW case, we estimate the diffusivity in the periarteriolar wall as:

$$D_{wall} = D_{ECS} \frac{0.3\% \;of\;wall\;is\;endfeet\;gaps}{20\% \;void\;volume\;ECS} \frac{{24\;{\text{nm}}\; endfeet\;gaps}}{{60\;{\text{nm}}\;ECS\;gaps }} = 0.6\% \;D_{ECS}$$

It is not computationally feasible to refine the mesh to resolve the 1.5 μm thickness of the endfeet, therefore an equivalent mass-transfer resistance (*L/D*) is used—a higher diffusivity for a longer distance:

$$D_{wall}^{\prime} = D_{wall} \frac{{12.5\;\upmu {\text{m}} \;chosen\; wall \;thickness}}{{1.5 \;\upmu {\text{m}} \;actual\; wall\; thickness}} = 5\% \; D_{ECS} \; \left( {for \;12.5 \;\upmu {\text{m}} \;wall\; thickness} \right)$$

It has been proposed that the perivenular wall is “looser” with respect to the transport of solutes than the periarteriolar wall [38], so we choose $D_{\text{arteriolar wall}}^{{\prime }}$ = 5% *D*_*ESC*_ and $D_{\text{venular wall}}^{{\prime }}$ = 10% *D*_*ESC*_. For the loose PVW case, $D_{wall}^{{\prime }} = D_{ECS}$. A no-flux boundary condition is applied to all other boundaries. Initial conditions differ depending on the physical situation being simulated and are given below. Apparent diffusivity is assumed to be homogeneous and isotropic.

In RTI experiments, a current is applied to the probe, creating a source of molecules at the probe’s insertion point. The RTI probe is represented as a point source, an assumption that is consistent with previous analysis of RTI data [14]. The source magnitude is derived from Faraday’s law: $s = \left( {I/F} \right) \cdot \left( {M/z} \right) \cdot n_{t}$, where $n_{t}$ is an experimentally measured probe efficiency. Concentration versus time is measured at a detection point 150 μm from the source. Experimental variability among replicates is of key interest in the present work. When executing an RTI experiment, the probes are inserted with very limited knowledge of neighboring arteriole and venule locations. Therefore, to simulate experimental variability, seven random source point locations are chosen within the center 195 µm × 195 µm × 195 µm of the domain. A solution is generated for each source point, and curves of concentration vs. time are recorded for 16 detection points surrounding each source point at a distance of 150 µm. The exterior boundaries have been placed far enough from the source to have little effect (this was tested by varying the domain size), so the no-flux boundary condition is sufficient. Initially, solute concentration is $c = 0$ throughout the domain. TMA free (unhindered) diffusivity (*D*) is 1.3 × 10^−5^ cm^2^ s^−1^ [14]. For RTI experimental data used for comparison to simulations, subjects were anesthetized, using urethane for Cserr experiments and ketamine/xylazine for Xie and Kress.

The clearance simulation, which is symmetrical in the axial direction of the vessels, utilizes a two-dimensional model that looks exactly like the slice shown in Fig. 3. An initial uniform concentration of soluble Aβ is applied to the interstitium and its concentration tracked over time for various conditions. Aβ diffusivity is estimated based on the free diffusivity of Dextran 3, *D* = 2.3 × 10^−6^ cm^2^ s^−1^, with a tortuosity of 2.04 [20].

The resulting system of partial differential equations is solved using FEniCS [41, 42]. The time-derivative is discretized using a backward difference (i.e., an implicit method). The finite element meshes on which the computations are performed are generated using CGAL [43]. The bulk of the simulations were performed on a mesh consisting of over 880,000 tetrahedral elements. The accuracy of the results was tested by (1) decreasing the time step by half and, separately, (2) approximately doubling the number of mesh elements; each resulted in less than a 1% variance. Post-processing of simulation data is carried out using Excel and Paraview.

### Model parameters and variables

Parameters and variables used in the model along with their values, or value range, and references are reported in Table 2. Many previous models of transport in the brain required a number of assumptions to obtain a simple enough model that an analytical solution is available. We have purposefully sought to minimize the number of assumptions and adjustable variables to examine a specific hypothesis, bulk flow. For the model presented in this paper, some assumptions are more likely to be correct than others. For example, the values used for free diffusivity, void volume, and distance between vessels are all based on extensive experimental measurements and are likely to be relatively accurate. For variables like these where we are confident in the assumptions made, we use the values given in Table 2 and those values are not varied significantly in the analysis of the model predictions. For other variables, notably the pressure difference between the periarteriolar wall and the perivenular wall, there is far more uncertainty so a large range of values is explored, and then model predictions are compared to experimental measurements.

## Results

### Interstitial bulk-flow simulations

**Fig. 4:**
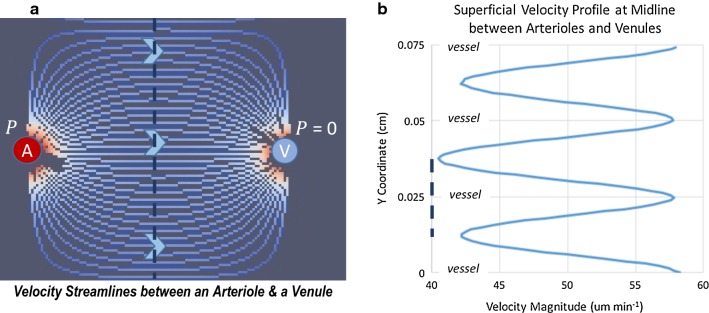
Superficial velocity streamlines and velocity profile for $v$ = 50 μm min^−1^. **a** Streamlines show how flow is organized from the arteriole to venule; this pattern repeats between arteriole and venule pairs throughout the domain. **b** Velocity profile at the midline slice of the domain at an instant in time coinciding with the average arteriolar pressure. Note velocity is highest in a direct line between an arteriole and a venule

Bulk-flow simulations were performed for a range of pressures, assuming both the hydrostatic and hydrodynamic cases (see “Background”), and the range of hydraulic conductivities found in the literature. For the hydrostatic case, a pressure of 0.2 mmHg is used. A maximum hydrodynamic pressure difference of 1–10 mmHg is used (the same range is explored by Jin [38]), based on 1–10% of systolic arteriolar pressure, which is approximately 65–100 mmHg. The resulting bulk-flow velocity varies with space and time; Fig. 4 shows example velocity streamlines between an arteriole and a venule and an instantaneous velocity profile across the midline slice of the domain. Velocity is highest in a direct line between arteriole and venule, but only varies ± 18% from the average. Table 3 reports the average bulk-flow superficial-velocity calculated from flow simulations for the range of hydraulic conductivities and pressures. To readily compare different conditions, the velocity is averaged over time and the entire domain. A bulk flow superficial-velocity of 0.5–25 μm min^−1^ (0.1–4 × 10^−4^ cm s^−1^) results from mid-range hydraulic conductivity and the range of pressures. This corresponds to a superficial volumetric flow rate of 0.05–2.4 μL g^−1^ min^−1^ (for brain tissue density = 1.0425 g cm^−3^).

**Table 3:** Simulation results for bulk-flow superficial-velocity in the brain interstitium

Hydraulic conductivity (cm^2^ mmHg^−1^ s^−1^)	Average bulk-flow superficial-velocity (μm min^−1^)			
	For P_avg_ = 0.2 mmHg	For P_max_ = 1 mmHg P_avg_ = 0.8 mmHg	For P_max_ = 3 mmHg P_avg_ = 2.4 mmHg	For P_max_ = 10 mmHg P_avg_ = 8 mmHg
2 × 10^−6^ [38, 45]^a^	5	25	75	250
2 × 10^−7^ [46]	0.5	2.5	7.5	25
2 × 10^−8^ [35]	0.05	0.25	0.75	2.5

Volume-averaged (over full domain), time-averaged velocity is reported for each condition. A range of hydraulic conductivity values from the literature are reported and used in the simulation. A range of periarteriolar wall pressures are used, and further described in the text. Wall pressure is an oscillatory function in the model; maximum and mean pressure are reported in the table

^a^Reports value of 1.2 × 10^−6^ cm^2^ mmHg^−1^ s^−1^, for parenchyma only based on simulation

### Simulations of real-time iontophoresis experiments

Comparison of simulations to RTI experimental data is used to test theories for mechanisms of interstitial transport in the brain: diffusion, convection, perivascular exchange, and conditions at the perivascular wall. In addition, the sensitivity of results to sources of experimental variability, vessel separation, and velocity magnitude are investigated. A list of transport simulations performed and a summary statistical analysis comparing the simulations to the experimental values is given in Tables 4 and 5.

**Table 4:** Summary of simulations and sensitivity analysis performed

Description	$C_{max,mean}$ (mM)	Range
Experimental	1.1	0.70
Simulation base case: diffusion only in homogeneous tissue (no perivascular exchange)	1.2	0.0
Estimates of range contribution from experimental variability		
Inter-animal and intra-animal tissue variation: void volume (± 0.005)	1.2	0.12
Inter-animal and intra-animal tissue variation: tortuosity (± 0.05)	1.2	0.09
Error in probe separation distance (± 2 μm)	1.2	0.02
Other factors (e.g., tissue damage)	1.2	Unknown
Diffusion only plus est. experimental variability	1.2	0.23
Additional transport mechanisms (convection, perivascular exchange)		
Diffusion with perivascular exchange (λ = 1.85) Varying vessel separation (distance in parentheses)	1.25 (225 μm) 1.28 (250 μm) 1.31 (275 μm)	0.44 (225 μm) 0.47 (250 μm) 0.42 (275 μm)
Diffusion and convection ($v$ = 50 μm min^−1^) in homogeneous tissue (no perivascular exchange)	1.2	0.24
Diffusion and convection with perivascular exchange (λ = 1.85) Varying bulk-flow velocity (in parentheses)	1.2 (10 μm min^−1^) 1.2 (50 μm min^−1^) 1.2 (100 μm min^−1^) 1.0 (250 μm min^−1^)	0.5 (10 μm min^−1^) 0.7 (50 μm min^−1^) 0.9 (100 μm min^−1^) 1.7 (250 μm min^−1^)

Using Cserr et al. [30] experimental case and base simulation conditions of α = 0.18, λ = 1.6, diffusion only in homogeneous tissue (no perivascular exchange routes). Simulations with perivascular exchange use λ = 1.85 for reasons described below

**Table 5:** Summary of boundary condition sensitivity analysis

Description	$C_{max,mean}$ (mM)	Range
Experimental	1.1	0.70
Boundary conditions		
Tight perivascular walls ($D_{wall} = 5\% D_{ECS}$)	1.2	0.66
Loose perivascular walls ($D_{wall} = D_{ECS}$)	0.86	1.17
Loose perivascular walls, no flux condition on arterioles ($D_{wall} = D_{ECS}$)	1.2	0.75

For boundary condition sensitivity, base case is diffusion and convection (${\text{v}}$ = 50 μm min^−1^) with perivascular exchange using vessel separation = 250 μm

As discussed in the introduction, many sources of variability are inherent to RTI experiments. We begin by attempting to quantify some of these sources of variability, namely inter-animal variation, tissue heterogeneity, and probe separation; others, such as tissue damage and the physiological state of the animal under study, are difficult to estimate. The tissue is simplistically characterized by α and λ, therefore the sensitivity of the simulation results to changes in these values was explored. Void volume between different experimental studies varies by at most 0.01 for the same general layer of the cortex, and tortuosity by 0.05 (Table 1). Table 4 reports this maximum variability due to tissue variation to have a combined range of 0.21. An error in probe separation measurement of 2 μm, results in a range of 0.02. Since diffusion-only simulations result in a range of zero, the same concentration curve in all directions independent of source location, the base case of diffusion-only plus the estimate of experimental variability is 0.23—about one-third of the observed experimental range.

**Fig. 5:**
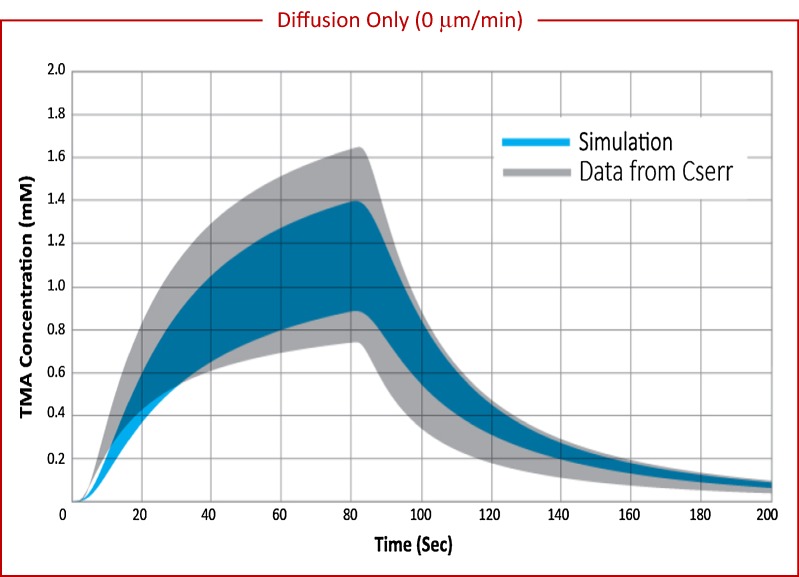
Range in TMA concentration versus time curves for experimental data compared with diffusion-only with perivascular exchange simulations. Experimental data from Cserr reported in grey (n = 33) [30] compared to diffusion-only simulations reported in blue (n = 112). Experimental median values were α = 0.18, and λ = 1.6. For simulation, $v$ = 0 μm min^−1^, α = 0.18, and λ = 1.85, vessel separation = 250 μm. Variability in the simulation is due to tissue heterogeneity introduced by discrete perivascular exchange locations within the domain, accounting for about two-thirds of the range observed experimentally

Diffusion only with perivascular exchange was simulated over a range of vessel separation (225–275 μm). Discrete locations where solute molecules leave the interstitium, at the PVW of the vessels penetrating the domain, contributes significantly to range by adding heterogeneity to the tissue. Perivascular exchange results in a range of 0.42–0.47 depending on vessel separation (Table 4), equivalent to about two-thirds of the range observed experimentally. C_max, mean_ increases with vessel separation, but no correlation is observed between vessel separation and range. The variability in range with vessel separation is likely due to small changes in the proximity between detection points and vessel locations. Figure 5 shows the range in concentration curves for a simulation with diffusion only and perivascular exchange (blue) compared to experimental data from Cserr (grey). The simulation results agree well in magnitude and shape with concentration curves from TMA-RTI experiments, but the range does not span the full experimental variability.

**Fig. 6:**
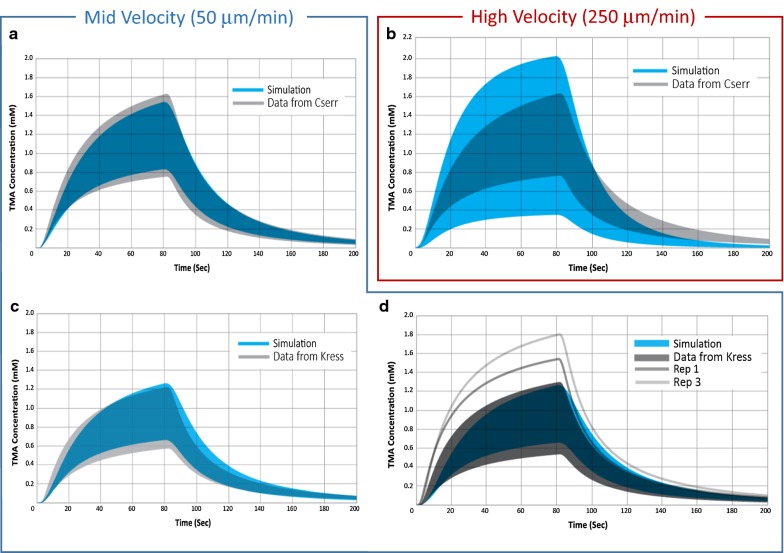
Range in TMA concentration curves for experimental data compared with diffusion and convection simulations with perivascular exchange. Simulations performed at a mid (50 μm min^−1^) and high (250 μm min^−1^) velocity based on bulk flow estimates. **a** Experimental data in rats from Cserr et al. (grey, n = 33) [30] compared with diffusion and mid-velocity convection simulations (blue, n = 112). Experimental median values were α = 0.18, and λ = 1.6, assuming diffusion only. For simulation, $v$ = 50 μm min^−1^, α = 0.18, and λ = 1.85. **b** Experimental data from Cserr et al. (grey, n = 33) [30] compared with diffusion and high-velocity convection simulations (blue, n = 112). For simulation, $v$ = 250 μm min^−1^. **c** Experimental data in mice from Kress et al. (grey) for female (n = 9) [25] compared with mid-velocity simulations (blue). Experimental median values were α = 0.224, and λ = 1.6, assuming diffusion only. For simulations, average bulk flow velocity = 50 μm min^−1^, α = 0.224, and λ = 1.85. **d** Experimental data in mice from Kress et al. (grey) for male (n = 11) [25] compared with mid-velocity simulations (blue). Experimental and simulation parameters same as **c**. The range for 50 μm min^−1^ simulation results is equivalent to the full variability reported by both Cserr et al. and Kress et al. consistent with the presence of bulk flow. Range for the 250 μm min^−1^ simulation is much higher than experimental observations, suggesting that bulk flow in the interstitium is significantly less than 250 μm min^−1^

Diffusion and convection simulations were performed for a range of bulk-flow velocity, with and without perivascular exchange. Convection of 50  μm min^−1^ without perivascular exchange gives a range of 0.24. When perivascular exchange is included in the simulation, the range increases to 0.7. In Fig. 6a, the range of concentration curves for simulations performed with an average bulk velocity of 50 μm min^−1^ and perivascular exchange (blue) is compared to the range in Cserr’s data (grey). Simulations performed for various source-detection path orientations (see “Methods”) relative to the flow field reflect the dependence of concentration curve upon orientation with the flow field, and result in significant range across simulation replicates. The range generated by a convective superficial velocity of 50 μm min^−1^ combined with diffusion and perivascular exchange is equivalent to the full experimental range reported by Cserr.

Figure 6b shows the range of simulated concentration curves for an average bulk flow velocity of 250 μm min^−1^ (blue) compared with Cserr’s data (grey, same as Figs. 5, 6a). At flow rates of 250 μm min^−1^ and above, the range is extremely high, and does not agree with reported experimental observations.

Similar results are observed when we analyze the data from Kress et al. [25] for male and female healthy, young adult mice. Simulation results for diffusion-only and a high bulk-flow velocity of 250 μm min^−1^, both with perivascular exchange, differ from experimental variability observations, similar to the Cserr data. In Fig. 6c, d, the range of concentration curves for simulations performed with an average bulk velocity of 50 μm min^−1^ (blue) is compared to the range in the Kress data (grey). Again, the range calculated from the simulation results accounts for the full variability in the experimental data for the female population. The two highest replicates from the male experimental data lie outside the range predicted by simulation. These high experimental replicates may have suffered from other sources of variability.

In the introduction, conflicting EM results regarding “tight” or “loose” endfoot arrangements at the perivascular wall were discussed. For the simulation results presented preceding this paragraph, a tight model was used, with the perivascular wall presenting a resistance to mass transfer greater than the ECS (see “Methods”). Simulations were also performed for a loose perivascular wall where $D_{wall} = D_{ECS}$—the resulting concentration curves have a significantly lower $C_{max,mean}$ = 0.86 and much greater range = 1.17 than the experimental data, $C_{max,mean}$ = 1.1 and range = 0.7 (Table 5). If the boundary condition is further changed such that material is only permitted to leave through the venular PVW (no exchange through the arteriolar PVW), then there is better agreement with the experiment, $C_{max,mean}$ = 1.2 and range = 0.75 for the simulation (Table 4). One would expect similar results if the vessels were further apart and both exchange routes were available.

Is it possible that flow is induced by the RTI experiment, and not physiological? Although the RTI experiment is designed to avoid electro-osmosis, it is possible that some occurs. Electro-osmosis means that instead of only TMA cations entering the brain tissue, solvent from the micropipette solution enters as well, generating a bulk flow. To understand the upper limit of the effect of electro-osmosis, a worst-case calculation was made assuming all of the TMA was delivered as the micropipette solution instead of as TMA cations alone. This worst case induced a bulk flow of only 0.6 μm min^−1^ at a distance of 150 μm from the source, a small fraction of the velocities discussed here.

The best agreement between simulations and experimental data results from a simulation tortuosity of 1.85, which is greater than the typical experimentally obtained value of 1.6. A higher tortuosity (λ) means a lower apparent diffusivity ($D^{*}$), as $D^{*} = D/\lambda^{2}$. In traditional RTI analysis, which assumes diffusion only, all transport mechanisms are lumped into this single variable, the apparent diffusivity. By overlooking other phenomena effecting transport—losses to perivascular exchange and convection—transport rates of all mechanisms are essentially combined into the single apparent diffusivity, increasing its magnitude and decreasing λ. In contrast, the simulation distinctly separates both convection and losses through perivascular spaces from diffusive transport in the interstitial tissue. This separation of mechanisms in the simulation means the apparent diffusivity now represents only the diffusional transport and it is therefore lower relative to diffusion-only RTI analysis. This was confirmed by performing simulations in a homogenous material, with no perivascular exchange, for which the best fit for the data was given by the experimental value for tortuosity (usually λ = 1.6).

It was shown above that a bulk flow velocity of $v$ = 50 μm min^−1^, with perivascular exchange, gives a range corresponding to the full experimental variability. However, if other sources of experimental variability are included, such as inter-animal tissue variation, a lower velocity would give better agreement. Therefore, for the following sections, we use a superficial bulk-flow velocity of $v$ = 15 μm min^−1^ to represent a more conservative estimate of $v$ considering contributions from the other sources of experimental variability.

### Implications for large-molecule transport

TMA is a small molecule (114 Da) with a relatively fast diffusivity. Molecules of interest in brain transport, such as Aβ (4.5 kDa) and tau (45 kDa), which are thought to play a significant role in neurodegenerative pathologies, are larger and have slower diffusivities. The Péclet number ($Pe$) is a ratio of convective to diffusive transport rates:

$$Peclet\;Number\;\left( {Pe} \right) = \frac{rate \;of\;convection}{rate\;of\;diffusion} = \frac{Lv}{D}$$

$Pe$ allows comparison of the relative importance of convection to diffusion for molecules with different apparent diffusivities. If transport is predominantly diffusion, then $Pe \ll 1$, and if transport is primarily bulk flow, $Pe \gg 1$. For interstitial transport, solutes move through three “materials” with differing diffusivities: periarteriolar wall, brain interstitium, and perivenular wall. To account for all materials, a mass-transfer resistance in series model is used where:

$$\begin{aligned} \frac{L}{D} \left( {overall} \right) & = \sum \frac{L}{D} = {\raise0.7ex\hbox{${L_{art. wall} }$} \!\mathord{\left/ {\vphantom {{L_{art. wall} } {D_{art. wall} }}}\right.\kern-0pt} \!\lower0.7ex\hbox{${D_{art. wall} }$}} \\ & \quad + {\raise0.7ex\hbox{${L_{ECS} }$} \!\mathord{\left/ {\vphantom {{L_{ECS} } {D_{ECS} }}}\right.\kern-0pt} \!\lower0.7ex\hbox{${D_{ECS} }$}} + {\raise0.7ex\hbox{${L_{ven. wall} }$} \!\mathord{\left/ {\vphantom {{L_{ven. wall} } {D_{ven. wall} }}}\right.\kern-0pt} \!\lower0.7ex\hbox{${D_{ven. wall} }$}} \end{aligned}$$

**Fig. 7:**
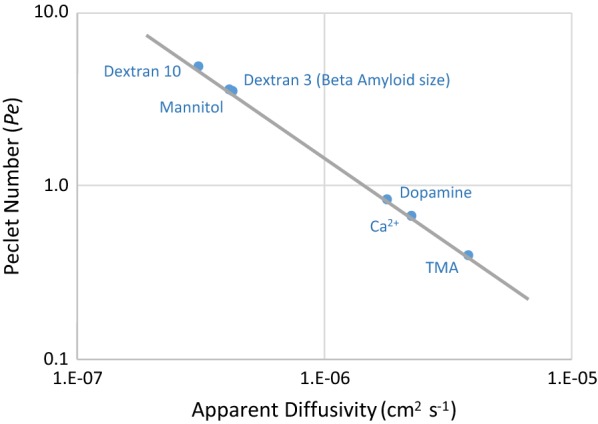
Péclet number versus apparent diffusivity for various molecules of interest in brain transport. L = 250 μm, $v$ = 15 μm min^−1^, and apparent diffusivity (D^*^) specific to each molecule. $Pe = v$ L/D^*^ is the ratio of convective to diffusive transport rates. For $Pe \approx 1$, diffusive and convective rates are balanced; for $Pe > 1$, convection exceeds diffusion. The graph shows for $v$ = 15 μm min^−1^ bulk flow is not large enough to be dominant and not small enough to be ignored

Figure 7 reports Péclet numbers for molecules relevant to brain transport as a function of their apparent diffusivity for a bulk flow of $v$ = 15 μm min^−1^. Tortuosity for the molecules other than TMA were measured by IOI [20] or radiotracer techniques [14] and adjusted for the tortuosity used here for the brain interstitium only.

As expected, TMA has a Péclet number less than 1 ($Pe \approx 0.4$), indicating its interstitial transport is diffusion-dominant. Therefore, TMA is an appropriate molecule for probing the structure of brain tissue using a diffusive-transport assumption. However, Dextran-3 kDa (Dex3), similar in size to Aβ, has a Péclet number of 4, meaning convection will have an effect similar in magnitude to, or potentially greater than, diffusion within the brain tissue. Many molecules of interest to brain pathologies are even larger than Dex3, therefore, the magnitude of convective transport due to bulk flow is likely to be of similar or greater magnitude than diffusive transport. It follows that bulk flow should be considered when studying large-molecule transport in the brain.

### Clearance simulations

The previous discussion focused on the transport properties of brain tissue. Now we explore how these properties impact the efficiency of clearing materials from brain tissue. Using the findings of previous sections, simulations of Aβ clearance were performed to investigate the impact of possible convective bulk flow on metabolite clearance. Iliff et al. report data for clearance of an interstitial injection of radiolabeled Aβ from the entire brain for aquaporin-4 (*Aqp4*) null and WT mice [4] (AQP4 is a water transport channel localized to the astrocyte endfeet, Fig. 1). As the model presented here is of a small volume of the interstitium and it will be compared to data taken for the whole brain, an assumption is being made that transport through the interstitium is the rate-limiting step in molecular clearance. This is not known to be true, however, the interstitium does represent the smallest spaces in which extracellular transport is occurring. Calculations made using this assumption will result in a conservative assessment of transport rate through the interstitium as several processes are being ignored. None-the-less it seems an instructive exercise for testing our outcomes.

**Fig. 8:**
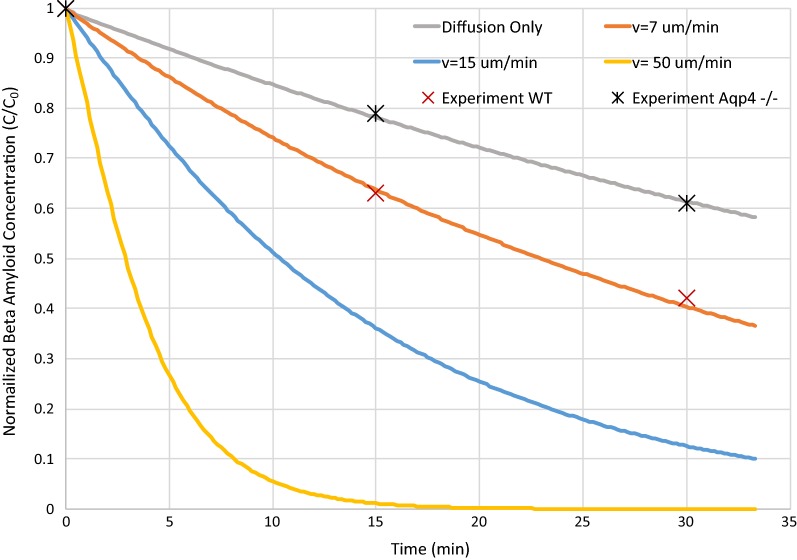
Aβ clearance from interstitial injection, experimental data compared with simulations. Experimental data from Iliff for *Aqp4* KO and WT mice [4]. Simulation results at various bulk-flow rates and for diffusion only. Details of simulation described in “Methods”. Periarteriolar wall and perivenular wall diffusivities are 2.5% and 5% of interstitial diffusivity respectively, to fit experimental data for *Aqp4* null mice (which are hypothesized to have no bulk flow in the interstitium). Based on conservative assumptions, simulations for a bulk-flow velocity of 7 μm min^−1^ fit the experimental data for WT mice

Assuming the absence of bulk flow in the *Aqp4* null mice, a diffusion-only simulation (Fig. 8) predicts perivascular wall diffusivities of $D_{\text{arteriolar wall}}^{{\prime }}$ = 2.5% *D*_*ESC*_ and $D_{\text{venular wall}}^{{\prime }}$ = 5% *D*_*ESC*_—half those used above for TMA. It is reasonable to expect higher tortuosity for a larger molecule within the tight perivascular walls. Using these wall diffusivities, simulations were performed for various interstitial pressure differences resulting in various bulk-flow velocities. A simulation for *v* = 7 μm min^−1^ shows best agreement with experimental data for the WT mice (Fig. 8). It should be noted that a bulk-flow rate of zero in the *Aqp4* null mice is unlikely to be true as water transport also occurs through gaps in astrocytic endfeet; therefore, the fit presents a conservative calculation of bulk-flow velocity, and higher bulk-flow velocities are possible. Further, simulations show bulk flow to have a significant impact on clearance of Aβ, even at low velocities (Fig. 8).

## Discussion

This work compares the range in simulated TMA-RTI concentration curves inherent to different transport mechanisms to experimental range to show evidence of (1) interstitial convective flow and (2) perivascular exchange. Experimental range will be comprised of contributions from several sources, which are likely to interact in ways that are not purely additive. However, identifiable sources were investigated separately in an attempt to quantify their relative contributions. The simplest case of diffusion only in a homogeneous medium has no variability with source or detection points, and therefore results in a range of zero. The contribution of tissue variation between experimental subjects and within an individual subject to range was estimated based on differences in void volume and tortuosity between experimental data sets and found to be 0.23 (about one-third of the full experimental variability of 0.70). Additional sources of experimental variability, such as tissue damage, are also potentially present but not possible to quantify. This leaves us with approximately two-thirds of the full experimental variability that may be caused by transport mechanisms not included in the experimental data analysis.

Simulations attribute a relative range of 0.42–0.47 to diffusion and perivascular exchange for vessel separation ranging from 225 to 275 μm. The boundary-condition assumption of a zero solute concentration in the perivascular space is likely extreme. Asgari predicts perivascular concentrations of about 30% of the tissue concentration approximately 20 min following interstitial injection [15], for a model assuming dispersive transport in the perivascular space. A model assuming perivascular convection may predict lower perivascular concentrations, but likely not zero. A more realistic perivascular concentration would result in a lower range attributable to perivascular exchange. In addition, range due to perivascular exchange is likely to be dependent on the arrangement of the arterioles and venules, which were not varied in this work, making higher or lower range contributions possible.

Simulations also show that the presence of convection can contribute significantly to range, depending on the magnitude of the bulk-flow velocity, with a contribution of 0.24 at $v$ = 50 μm min^−1^. When all transport mechanisms are combined, and quantifiable experimental variability sources are added, the resulting range matches the experimental variability for $v$ = 10–50 μm min^−1^. Similar interstitial bulk-flow superficial velocities have been reported in the literature: Abbott et al. estimated 10 μm min^−1^ in the cuttlefish brain [47]; Rosenberg et al. measured 10.5 μm min^−1^ in white matter [48]; however, Holter et al. calculate a much lower bulk flow velocity around 0.3 μm min^−1^ [35]. The shape of the simulated concentration curves for the combination of all transport mechanisms also agrees well with the experimental curves, although simulated curves deviate from experimental-fit curves over the first 5 s of the RTI experiment. Understanding this difference may help identify relevant transport mechanisms not accounted for currently. Even though it is difficult to say the exact proportions of sources and mechanisms that comprise the full experimental range, at a minimum it has been demonstrated based on this analysis of RTI data that the presence of bulk flow cannot be excluded.

Simulations of Aβ clearance calculate a conservative bulk flow velocity $v$ = 7 μm min^−1^. This estimate includes the conservative assumptions of no ISF flow in *Aqp4* KO mice and transport across the ECS as the only step in the complex process of transport through the entire brain; and may therefore be considered a lower limit for bulk-flow velocity. Smith et al. found no difference in clearance between WT and *Aqp4* KO mice. However, Mestre et al. demonstrated the choice of anesthesia and methods of tracer injection employed by Smith suppress CSF influx [6]. Mestre’s work includes a meta-analysis citing decreased ISF tracer clearance in *Aqp4* KO mice and rats in five of six studies (the one outlier being Smith et al.).

Asgari et al. suggests the importance of separating fluid and solute pathways in predicting clearance for *AQP4* null animals [15], as the fluid has access to additional transport pathways across the PVW. In the simulations presented here fluid and solute pathways through the perivascular wall are treated separately. The model assumes the solute may enter or exit the interstitium only through the gaps between astrocytic endfeet. Two cases are considered: (a) a tight case, based on the work of Mathiisen and (b) a loose case, based on the work of Korogod. In each case, the gap widths and the percent of the surface covered by gaps is used to calculate a PVW diffusivity for the solute that is a fraction of its ECS diffusivity. In contrast, the fluid will theoretically move both through the gaps between astrocytic endfeet and through aquaporin channels. However, to calculate fluid velocity, the model assumes a pressure just inside the interstitial space, estimated as a small percentage of arteriolar pressure. Therefore, fluid pathways through the PVW are not specifically considered in the model, except to the extent that a reduction in pressure across the wall is taken into consideration when estimating a reasonable pressure range to explore.

It may be possible to further investigate the presence or absence of interstitial convection through comparison to experiments where any potential physiological flow has ceased. Physiological flow is ceased in brain slice experiments, where reported tortuosity is higher than in vivo experiments for the same region of the brain (Table 1) indicating slower transport than with physiological flow present. Brain slice experimental-replicate data present an opportunity that could be pursued in the future. However, brain slice experiments pose additional sources of variability not present during in vivo experiments, e.g., water uptake during incubation and loss of TMA from the slice surface that is not accounted for by conventional analysis [29]. The additional sources of variability would need to be quantified for a useful comparison.

Comparison of simulation to experimental range supports the possibility of interstitial bulk-flow velocity on the order of 10 μm min^−1^–an outcome independent of the origin of said flow. Based on an intermediate value for hydraulic conductivity, such a flow rate requires an average pressure difference of around 2–5 mmHg. These findings are consistent with Jin [38], who reported “significant convective transport requires a sustained pressure difference of several mmHg”. A 2–5 mmHg pressure magnitude requires hydrodynamic pressure, but leaves outstanding the question of how much of the arteriolar pressure wave (with a peak pressure between 65 and 100 mmHg) is translated beyond the vessel wall. Pressure generated in the periarterial space by arteriolar pulsation is a hypothesis for which there is conflicting support [15, 16, 34]. However, as long as the vessel wall is not completely rigid, a small fraction will be translated and the exact amount of this translation is thus an important area of further investigation.

The interstitial bulk-flow velocity $v$ = 10 μm min^−1^ can also be expressed as a volumetric flow rate of 1.0 μL g^−1^ min^−1^. Hladky’s impressive review of clearance of specific substances from the brain interstitium calculates a perivascular flow rate of 0.6–1.2 μL g^−1^ min^−1^ based on observations of inulin and sucrose clearance from brain tissue [7] (although Hladky notes the calculated perivascular rate exceeds current estimates of CSF production rate, 0.25 μL g^−1^ min^−1^, and is unlikely to be made up by fluid secretion from the BBB). If the link between periarterial and perivenular flow is bulk flow across the interstitium, then the interstitial flow rate would also have to be around 1 μL g^−1^ min^−1^ due to continuity of mass—consistent with conclusions from the work presented here.

Transport conditions at the perivascular wall were investigated, with the best fit resulting from a tight wall assumption, based on Mathiisen [10]. In the simulation where perivascular wall diffusivity did not differ from ECS diffusivity, based on Korogod [13], less TMA accumulation due to faster transport at the PVW resulted in low $C_{max,mean}$ = 0.86 mM and a large range = 1.17, compared to the experiment $C_{max,mean}$ = 1.1 mM and range = 0.7. Thus simulations support a mass-transfer resistance at the PVW, and further work is necessary to clarify the details of the PVW resistance.

This work focused on RTI experimental data due to its quantitative nature and accessibility; additional information may be gleaned by investigating IOI and magnetic resonance imaging (MRI). Although IOI experimental data is complex to analyze and not directly comparable to simulation (as described in “Background”), comparison of concentration simulations to intensity measurements may still provide useful insights into mechanisms of transport, particularly for larger molecules. MRI, which enables studies of the entire brain, is a promising field, especially as image resolution improves (MRI can currently resolve in the sub-millimeter range; resolution of microns is required to measure interstitial bulk flow). Contrast-enhanced MRI data following the transport of tracers from the cisterna manga into brain interstitium has been reported in rats [49, 50]. MRI images have the additional benefit of also containing key anatomical features, which may provide accurate and specific information such as vascular arrangement and dimensions that are currently estimated (Additional file 1).

## Conclusions

In conclusion, the analysis described here, comparing transport simulations to previously published experimental data, supports that interstitial transport may occur by both diffusion and convection (bulk flow), with both mechanisms potentially relevant and the apparent diffusivity, related to molecular size, determining which is dominant. Simulations show that published RTI experimental range and tracer clearance studies allow for interstitial bulk-flow superficial velocities from $$v$$ = 7 to 50 μm min^−1^; corresponding to intrinsic velocities on the order of 100 μm min^−1^ ($$v_{i} = v/0.2)$$. A useful finding for the scientists developing approaches for evaluating slow interstitial bulk flow over long distances. Results also support (1) the hypothesis of perivascular space allowing exchange between the brain interstitium, the subarachnoid CSF, and perivenous drainage out of the brain; and (2) increased mass transfer resistance at the PVW (as compared to the ECS).

These findings are consistent with the prevailing conclusion of RTI experiments—transport of small molecules (such as those used in RTI) in the brain interstitium is well explained by a diffusion-dominant model; and RTI is an excellent technique for probing the structure of the extracellular space. However, the effect of bulk flow on solute transport increases with molecular size. For the large molecules of interest in neuropathology, bulk flow may be an important mechanism of transport. These molecules have small unhindered diffusivities, made even smaller when moving through the narrow spaces of tortuous extracellular space. Simulations of Aβ clearance from the brain, fitted to experimental data, show evidence for bulk flow and its enhancement of clearance rate. Further exploration of bulk flow in the brain interstitium, particularly its driving force, and its relevance to the transport of biologically important molecules is warranted. Even relatively small contributions from interstitial bulk flow may have a significant impact on molecular transport over the span of neurodegenerative disease progression.

## Additional files

**Additional file 1.** Supplementary results and discussion, including 1) simulations compared to RTI experimental results for asleep and awake states and 2) sensitivity of range to individual source-point location.** Figure S1:** TMA concentration curves for asleep and awake RTI experiments, comparing simulations to published experimental data.** Figure S2:** Dependence of TMA concentration-curve range on individual source-point location.

## Abbreviations

Aβ: beta amyloid
AQP4: aquaporin 4
BBB: blood–brain barrier
CSF: cerebrospinal fluid
Dex3: Dextran-3 kDa
ECS: extra-cellular space
EM: electron microscope
IOI: Integrative Optical Imaging
ISF: interstitial fluid
KO: knock-out
MRI: magnetic resonance imaging
*Pe*: Péclet number
PVS: perivascular space
PVW: perivascular wall
RTI: real-time iontophoresis
TMA: tetramethylammonium
WT: wild-type

## References

[1] Agnati LF, Bjelke B, Fuxe K (1992). Volume transmission in the brain. Am Sci.

[2] Selkoe DJ, Hardy J (2016). The amyloid hypothesis of Alzheimer’s disease at 25years. EMBO Mol Med.

[3] Iliff JJ, Chen MJ, Plog BA, Zeppenfeld DM, Soltero M, Yang LJ (2014). Impairment of glymphatic pathway function promotes tau pathology after traumatic brain injury. J Neurosci.

[4] Iliff JJ, Wang M, Liao Y, Plogg BA, Peng W, Gundersen GA (2012). A paravascular pathway facilitates CSF flow through the brain parenchyma and the clearance of interstitial solutes, including amyloid beta. Sci Transl Med.

[5] Smith AJ, Yao X, Dix JA, Jin B-J, Verkman AS (2017). Test of the ‘glymphatic’ hypothesis demonstrates diffusive and aquaporin-4-independent solute transport in rodent brain parenchyma. Elife..

[6] Mestre H, Hablitz LM, Xavier AL, Feng W, Zou W, Pu T (2018). Aquaporin-4-dependent glymphatic solute transport in the rodent brain. eLife..

[7] Hladky SB, Barrand MA (2018). Elimination of substances from the brain parenchyma: efflux via perivascular pathways and via the blood–brain barrier. Fluids Barriers CNS..

[8] Nicholson C, Hrabetova S (2017). Brain extracellular space: the final frontier of neuroscience. Biophys J.

[9] Hannocks MJ, Pizzo ME, Huppert J, Deshpande T, Abbott NJ, Thorne RG (2018). Molecular characterization of perivascular drainage pathways in the murine brain. J Cereb Blood Flow Metab.

[10] Mathiisen TM, Lehre KP, Danbolt NC, Ottersen OP (2010). The perivascular astroglial sheath provides a complete covering of the brain microvessels: an electron microscopic 3D reconstruction. Glia..

[11] Abbott NJ, Pizzo ME, Preston JE, Janigro D, Thorne RG (2018). The role of brain barriers in fluid movement in the CNS: is there a ‘glymphatic’ system?. Acta Neuropathol.

[12] Wolak DJ, Thorne RG (2013). Diffusion of macromolecules in the brain: implications for drug delivery. Mol Pharm.

[13] Korogod N, Petersen CCH, Knott GW (2015). Ultrastructural analysis of adult mouse neocortex comparing aldehyde perfusion with cryo fixation. Elife..

[14] Nicholson C (2001). Diffusion and related transport mechanisms in brain tissue. Rep Prog Phys.

[15] Asgari M, de Zelicourt D, Kurtcuoglu V (2016). Glymphatic solute transport does not require bulk flow. Sci Rep.

[16] Mestre H, Tithof J, Du T, Song W, Peng WG, Sweeney AM (2018). Flow of cerebrospinal fluid is driven by arterial pulsations and is reduced in hypertension. Nat Commun.

[17] Carare RO, Bernardes-Silva M, Newman TA, Page AM, Nicoll JAR, Perry VH (2008). Solutes, but not cells, drain from the brain parenchyma along basement membranes of capillaries and arteries: significance for cerebral amyloid angiopathy and neuroimmunology. Neuropathol Appl Neurobiol.

[18] Arbel-Ornath M, Hudry E, Eikermann-Haerter K, Hou S, Gregory JL, Zhao LZ (2013). Interstitial fluid drainage is impaired in ischemic stroke and Alzheimer’s disease mouse models. Acta Neuropathol.

[19] Hawkes CA, Jayakody N, Johnston DA, Bechmann I, Carare RO (2014). Failure of perivascular drainage of amyloid in cerebral amyloid angiopathy. Brain Pathol.

[20] Sykova E, Nicholson C (2008). Diffusion in brain extracellular space. Physiol Rev.

[21] Adams DL, Piserchia V, Economides JR, Horton JC (2015). Vascular supply of the cerebral cortex is specialized for cell layers but not columns. Cereb Cortex.

[22] Nishimura N, Schaffer CB, Friedman B, Lyden PD, Kleinfeld D (2007). Penetrating arterioles are a bottleneck in the perfusion of neocortex. Proc Natl Acad Sci USA.

[23] Nicholson C, Phillips JM (1981). Ion diffusion modified by tortuosity and volume fraction in the extracellular micro-environment of the rat cerebellum. J Physiol.

[24] Xie L, Kang H, Xu Q, Chen MJ, Liao Y, Thiyagarajan M (2013). Sleep drives metabolite clearance from the adult brain. Science.

[25] Kress BT, Iliff JJ, Xia M, Wang M, Wei HS, Zeppenfeld D (2014). Impairment of paravascular clearance pathways in the aging brain. Ann Neurol.

[26] Lehmenkuhler A, Sykova E, Svoboda J, Zilles K, Nicholson C (1993). Extracellular-space parameters in the rat neocortex and subcortical white-matter during postnatal-development determined by diffusion analysis. Neuroscience.

[27] Vorisek I, Sykova E (1997). Evolution of anisotropic diffusion in the developing rat corpus callosum. J Neurophysiol.

[28] Mazel T, Simonova Z, Sykova E (1998). Diffusion heterogeneity and anisotropy in rat hippocampus. NeuroReport.

[29] Kume-Kick J, Mazel T, Vorisek I, Hrabetova S, Tiao L, Nicholson C (2002). Independence of extracellular tortuosity and volume fraction during osmotic challenge in rat neocortex. J Physiol.

[30] Cserr HF, Depasquale M, Nicholson C, Patlak CS, Pettigrew KD, Rice ME (1991). Extracellular volume decreases while cell-volume is maintained by ion uptake in rat-brain during acute hypernatremia. J Physiol.

[31] Perezpinon MA, Tao L, Nicholson C (1995). Extracellular potassium, volume fraction, and tortuosity in rat hippocampal CA1, CA3, and cortical slices during ischemia. J Neurophysiol.

[32] Nicholson C, Tao L (1993). Hindered diffusion of high-molecular-weight compounds in brain extracellular microenvironment measured with integrative optical imaging. Biophys J.

[33] Cserr HF, Cooper DN, Suri PK, Patlak CS (1981). Efflux of radiolabeled polyethylene glycols and albumin from rat-brain. Am J Physiol.

[34] Iliff JJ, Wang M, Zeppenfeld DM, Venkataraman A, Plog BA, Liao Y (2013). Cerebral arterial pulsation drives paravascular CSF-interstitial fluid exchange in the murine brain. J Neurosci.

[35] Holter KE, Kehlet B, Devor A, Sejnowski TJ, Dale AM, Omholt SW (2017). Interstitial solute transport in 3D reconstructed neuropil occurs by diffusion rather than bulk flow. Proc Natl Acad Sci USA.

[36] Kyrtsos CR, Baras JS (2015). Modeling the role of the glymphatic pathway and cerebral blood vessel properties in Alzheimer’s disease pathogenesis. PLoS ONE..

[37] Khaled ARA, Vafai K (2003). The role of porous media in modeling flow and heat transfer in biological tissues. Int J Heat Mass Transf.

[38] Jin B-J, Smith AJ, Verkman AS (2016). Spatial model of convective solute transport in brain extracellular space does not support a “glymphatic” mechanism. J Gen Physiol.

[39] Kinney JP, Spacek J, Bartol TM, Bajaj CL, Harris KM, Sejnowski TJ (2013). Extracellular sheets and tunnels modulate glutamate diffusion in hippocampal neuropil. J Comp Neurol.

[40] Thorne RG, Nicholson C (2006). In vivo diffusion analysis with quantum dots and dextrans predicts the width of brain extracellular space. Proc Natl Acad Sci USA.

[41] Logg A, Mardal KA, Wells GN (2012). Automated solution of differential equations by the finite element method: the FEniCS book.

[42] Logg AW, Wells GN, Hake J. DOLFIN: a C ++/Python finite element library. lecture notes in computational science and engineering. In: Automated solution of differential equations by the finite element method, vol. 84. Berlin: Springer; 2012.

[43] Project TC. CGAL user and reference manual. 4.13 ed. In: Board CE, editor. 2018.

[44] Xiong B, Li A, Lou Y, Chen S, Long B, Peng J (2017). Precise cerebral vascular atlas in stereotaxic coordinates of whole mouse brain. Front Neuroanat..

[45] Smith JH, Humphrey JAC (2007). Interstitial transport and transvascular fluid exchange during infusion into brain and tumor tissue. Microvasc Res.

[46] Basser PJ (1992). Interstitial pressure, volume, and flow during infusion into brain-tissue. Microvasc Res.

[47] Abbott NJ (2004). Evidence for bulk flow of brain interstitial fluid: significance for physiology and pathology. Neurochem Int.

[48] Rosenberg GA, Kyner WT, Estrada E (1980). Bulk flow of brain interstitial fluid under normal and hyper-osmolar conditions. Am J Physiol.

[49] Iliff JJ, Lee H, Yu M, Feng T, Logan J, Nedergaard M (2013). Brain-wide pathway for waste clearance captured by contrast-enhanced MRI. J Clin Invest.

[50] Ding G, Chopp M, Li L, Zhang L, Davoodi-Bojd E, Li Q (2018). MRI investigation of glymphatic responses to Gd-DTPA infusion rates. J Neurosci Res.

