# Supplementary material for: Analysis of convective and diffusive transport in the brain interstitium
Source: Fluids Barriers CNS. 2019 Mar 6;16:6. doi: 10.1186/s12987-019-0126-9 (PMC6402182; doi:10.1186/s12987-019-0126-9)
Supplement: Supplementary file 1 — Additional file 1. Supplementary results and discussion, including 1) simulations compared to RTI experimental results for asleep and awake states and 2) sensitivity of range to individual source-point location. Figure S1: TMA concentration curves for asleep and awake RTI experiments, comparing simulations to published experimental data. Figure S2: Dependence of TMA concentration-curve range on individual source-point location. [file 12987_2019_126_MOESM1_ESM.docx]

**Supplemental Material**

***Sleep versus Awake Simulations.*** Xie et al. investigated differences in transport in the mouse brain during sleep, wakefulness and under anesthesia (1). Using TMA-RTI, a significant decrease in void volume was observed with wakefulness, and interestingly, no change in tortuosity. (Most TMA-RTI experiments are carried out on anesthetized subjects, which the authors showed to be equivalent to sleep.) As hydraulic conductivity is known to increase with void volume, these results suggest a potential increase in convective transport with sleep. A “striking increase in convective exchange of CSF with ISF” during sleep was observed concurrent with an increased rate of Aβ clearance (1). From their results, the authors hypothesize the activity of the glymphatic system must increase during sleep.

A recent study by Ma et al. (2) reported that compared awake to immobilized anesthetized animals, CSF tracer efflux in awake behaving mice was more rapid. While this study suggests the reduced penetration of CSF tracer into the waking brain observed in Xie et al. may be related to the rapid efflux of tracer out of the cranial compartment rather than changes in CSF-ISF exchange, it does not directly evaluate this exchange with an approach independent of CSF tracers. Moreover, this interpretation does not appear to explain the observation that the clearance of the inert tracer inulin *from* the brain parenchyma is likewise slowed in the waking brain.

To test the predictive ability of the model, the sleep/wake experiments from Xie et al. were simulated. The change in void volume measured by Xie et al. and a calculated proportional decrease in hydraulic conductivity with wakefulness are reported in Table S1. Figure S1 reports average simulation results (see Table S1 for simulation parameters) alongside mean experimental results, showing good agreement.

**Table S1**. TMA Transport Parameters used for Asleep vs. Awake Simulations. Experimental values are tortuosity=1.55 & void volume=0.23 asleep and 0.14 awake.

| State | Void Volume | Hydraulic Conductivity (cm^2^ mmHg^-1^ s^-1^) | Pressure_max_ (mmHg) | Velocity  ($\mu m {min}^{-1})$ | Tortuosity |
| --- | --- | --- | --- | --- | --- |
| Asleep | 0.23 | 2 x10^-6^ | 0.6 | 15 | 1.8 |
| Awake | 0.14 | 2 x10^-7^ | 0.6 | <1 | 1.8 |

**Figure S1**. Mean TMA Concentration curves for asleep and awake experiment (1) comparing simulation and experimental data. Parameter values for simulation reported in Table S1. Simulations agree well with experimental data, supporting the hypothesis of an increase in hydraulic conductivity and bulk flow during sleep.

***Range Dependence on Source Point Location.*** The RTI source and detection locations, relative to the flow field and the locations of perivascular exchange, have a significant impact TMA-concentration curve range. To demonstrate this dependence, Figure S2 shows the range in TMA-concentration curves for simulations querying 16 different detection points surrounding each single source point. When considering the contour map shown in Figure S2, it is important to remember that the range value reported at the source point is for detection points surrounding the source point at a distance of 150 μm. Moving along the y-axis, range is greatest where convection is greatest—in a line between the arteriole and the venule. Here, each source point will have detection points directly upstream and downstream in the highest flow-field, giving the greatest difference in TMA-Concentration curves and the greatest range. Moving along the x-axis, range is greater for source points nearer the arterioles than the venules. This x-directional asymmetry is also due to convection, as a contour map of simulations without convection shows the minimum in the center of the domain; the contour has been shifted in the direction of flow. In an RTI experiment, it is anticipated that several different source-detection point pairs will be tested; the ensemble of these pairs is most likely to have a greater range than the range for single source points reported here.

**Figure S2**. Dependence of TMA concentration-curve Range on Source Point Location. Range reported for 16 detection points surrounding each source point at a distance of 150 μm. The domain of the contour is the central cube inside 2 arteriole-venule pairs; the x and y-coordinates of the graph match the coordinates of the domain. The plane is perpendicular to the vessels; grey quarter-circles indicate the location of vessels, incorporating their radius (15 μm) plus the 12.5-μm-thick PVW. ‘A’ denotes the arterioles and ‘V’ denotes the venules. Simulation conditions are $v$ = 50 μm min^-1^, α = 0.18, and λ = 1.85, vessel separation = 250 μm. Range is greatest for source points located where convection is the highest and located closer to the arterioles than venules.

**References**:

1. Xie L, Kang H, Xu Q, Chen MJ, Liao Y, Thiyagarajan M, et al. Sleep Drives Metabolite Clearance from the Adult Brain. Science. 2013;342(6156):373-7.

2. Ma Q, Ries M, Decker Y, Mueller A, Riner C, Buecker A, et al. Rapid lymphatic efflux limits cerebrospinal fluid flow to the brain. Acta Neuropathologica. 2019;137(1):151-65.
